# Strokelike Episodes in *PMM-2* Carriers Differ from Those in Mitochondrial Disorders

**DOI:** 10.1055/s-0043-1771183

**Published:** 2023-07-14

**Authors:** Josef Finsterer

**Affiliations:** 1 Neurology and Neurophysiology Center, Vienna, Austria

We read with interest the article by Sreedevi et al who reported the case of a 12-year-old girl with a congenital disorder of glycosylation (CDG) due to the variant c.710C > T in the phosphomannomutase-2 ( *PMM2* ) gene. 1 The patient manifested phenotypically with developmental delay, cognitive impairment, generalized hypotonia, and paraplegia. 1 It was concluded that couples with infertility or miscarriage and a child with CDG should undergo molecular genetic testing. 1 The study is excellent but has limitations that should be discussed.

We disagree with the statement in the abstract that patients with CDG manifest with strokelike episodes (SLEs). The term SLEs should be restricted to patients with a mitochondrial disorder (MID). SLEs are a pathognomonic phenotypic feature of mitochondrial encephalopathy, lactic acidosis, and strokelike episodes (MELAS). SLEs occur in other syndromic and nonsyndromic MIDs as well but with a much lower frequency than in MELAS. There are also reports about SLEs in patients with disorders other than MIDs, but in these patients, the MID most likely remained undiagnosed.

SLEs have a morphological correlate on imaging known as strokelike lesion (SLL). 2 SLLs manifest with a distinct pattern on multimodal magnetic resonance imaging (MRI), including hyperintensity on T2, fluid attenuated inversion recovery (FLAIR), perfusion weighted imaging (PWI) and diffusion weighted imaging (DWI), and hypointensity on oxygen extraction fraction (OEF). 2 Characteristically, SLLs initially expand in size to reach a nadir and disappear thereafter or remain visible as a distinct defect. 3 SLLs are not confined to a vascular territory.

A limitation of the study is that no MRI of the cerebrum was reported. 1 Because the patient had developmental delay, mental retardation, and hypotonia, it is mandatory to perform cerebral MRI to assess if any structural lesion or other abnormality could explain the clinical central nervous system (CNS) abnormalities. We should also know whether or not the index patient ever developed an SLE/SLL.

There is a discrepancy between the case description describing “generalized hypotonia” and the discussion that describes the patient with “spastic diplegia of the legs.” 1 This is contradictory and should be solved. We should know whether or not the index patient had either hypotonia or hypertonia (spasticity).

The patient is described as someone with “inadequate speech and language.” 1 It would be interesting to know if the patient had aphasia, dysarthria, or both.

In a current study with 50 PMM2 patients, 83% had coagulation disorders. 4 We should therefore know if any of the coagulation parameters gave an abnormal result and if the index patient had thrombosis or a history of bleeding.

We should also know if hypotonia was due to CNS involvement, myopathy or neuropathy. Were there any indications for myopathy or affection of the peripheral nerves?

Overall, the interesting study has limitations that call the results and their interpretation into question. Addressing these issues would strengthen the conclusions and could improve the status of the study. Because PMM2 deficiency usually manifests phenotypically with a multisystem disease, affecting organs other than the brain, these patients should be prospectively investigated for multisystem diseases. Multisystem involvement may be subclinical or may manifest only mildly, particularly at onset of these diseases.
